# TNF inhibitors significantly attenuate the humoral immune response to COVID-19 vaccination in patients with rheumatoid arthritis

**DOI:** 10.1093/rap/rkad065

**Published:** 2023-07-26

**Authors:** Arne Schäfer, Magdolna S Kovacs, Anna Eder, Axel Nigg, Martin Feuchtenberger

**Affiliations:** Diabetes Zentrum Mergentheim, Bad Mergentheim, Germany; Medizinische Klinik und Poliklinik II, University Hospital Würzburg, Würzburg, Germany; Rheumatologie, MVZ MED BAYERN OST, Burghausen, Germany; Rheumatologie, MVZ MED BAYERN OST, Burghausen, Germany; Rheumatologie, MVZ MED BAYERN OST, Burghausen, Germany; Medizinische Klinik und Poliklinik II, University Hospital Würzburg, Würzburg, Germany; Rheumatologie, MVZ MED BAYERN OST, Burghausen, Germany

**Keywords:** TNF inhibitor, DMARD, arthritis, rheumatoid, SARS-CoV-2, COVID-19, vaccination, immunogenicity, humoral

## Abstract

**Objective:**

Several studies on the immunogenicity of vaccination against coronavirus disease 2019 (COVID-19) in patients with immune-mediated inflammatory diseases have evaluated the influence of DMARDs. The aim of the work presented here was to compare the humoral vaccine response after two vaccinations between patients with RA undergoing TNF inhibitor therapy and healthy controls.

**Methods:**

We assessed the humoral immune response, as measured by titres of neutralizing antibodies against the S1 antigen of severe acute respiratory syndrome coronavirus 2 (SARS-CoV-2), in patients with RA and anti-TNF treatment *vs*. controls without immunomodulatory medication. One hundred and seven fully vaccinated individuals were included at 6 ± 1 weeks after the second vaccination [BioNTech/Pfizer (72.9%), AstraZeneca (17.8%) and Moderna (9.3%)]. Immune responses in terms of antibody titres were compared between both subgroups with (*n* = 45) and without (*n* = 62) exposure to anti-TNF medication. The comparison was performed as a cross-sectional, single-centre study approach using non-parametric tests for central tendency.

**Results:**

Anti-TNF medication produced a significantly impaired humoral immune response to vaccination against COVID-19. The maximum immune response was detected in 77.4% of control patients, whereas this decreased to 62.2% in participants treated with TNF inhibitors (*P* = 0.045; effect size, *d* = 0.194). Patients on combination treatment (anti-TNF medication and MTX, 17 of 45 subjects in the treatment group) did not differ significantly regarding humoral immune response compared with patients on monotherapy with TNF inhibitors only (*P* = 0.214).

**Conclusion:**

TNF inhibitors significantly reduce the humoral response following dual vaccination against COVID-19 in patients with RA.

Key message

- Anti-TNF medication significantly impaired the humoral immune response to vaccination against COVID-19.

## Introduction

The influence of DMARDs on the success of vaccination and the possible need to interrupt DMARD therapy in connection with vaccination against COVID-19 in patients with immune-mediated inflammatory diseases (IMIDs) are still the subject of scientific debate [1–6]. Initially, data on the immunogenicity of vaccines against COVID-19 were based on case series with a limited number of patients, inhomogeneous cohorts or analyses of retrospective data. Therefore, it was not uncommon for different authors to report conflicting results. The situation was complicated further by the exclusion of patients with IMIDs and congenital or acquired immunodeficiency from large severe acute respiratory syndrome coronavirus 2 (SARS-CoV-2) vaccine registration trials [7, 8]. Based on the available literature, there is a consensus that in addition to immunomodulatory DMARD therapy, other factors, such as age, co-morbidities and the underlying inflammatory rheumatic disease itself, influence the success of vaccination [9–13]. Cell-targeted therapies, such as abatacept or rituximab, together with glucocorticoids, appear to have the most pronounced attenuating effect, especially with regard to the humoral response after vaccination against COVID-19 [14–17]. Targeted synthetic DMARDs (tsDMARDs), such as JAK inhibitors, and cytokine-targeted biological DMARDs (bDMARDs) appear to have a lesser effect on the humoral response to vaccination [18–20]. A reduced but overall protective humoral immune response has already been documented for TNF inhibitors in patients with RA following vaccination against pneumococci or influenza [21]. Recommendations for COVID-19 vaccination of patients with IMIDs were published very early by scientific societies, such as the EULAR or the ACR [22, 23]. The aim of the present study was to compare the humoral vaccination response after two injections between RA patients treated with TNF inhibitors and healthy controls.

## Methods

### Patient recruitment

A total of 107 double-vaccinated patients were enrolled prospectively and consecutively in a routine care setting. Inclusion criteria for the TNF inhibitor subgroup were a confirmed diagnosis of RA according to ACR-EULAR 2010, an age of ≥18 years, and written informed consent to participate in the study. Exclusion criteria were a relative or absolute contraindication to TNF inhibitor therapy, known intolerance to TNF inhibitors, prior use of rituximab, use of conventional synthetic DMARDs (csDMARDs) other than MTX in combination with TNF inhibitors, and a history of SARS-CoV-2 infection. The control arm of the study included people without RA or any type of inflammatory rheumatic disease. Control patients were diagnosed with OA of the hands and were not taking any immunomodulatory medication.

The evaluation time point was 6 ± 1 weeks after the second vaccine dose for each patient. Patient recruitment followed a prospective, single-centre, cross-sectional study design. The present study was conducted at the rheumatological outpatient clinic of MED|BAYERN OST Medizinische Versorgungszentren, Burghausen, Germany. Importantly, the study sample included two independent patient subgroups (with and without RA and anti-TNF medication). With respect to the objectives of the present study, the primary aim was to compare these two patient groups (with and without TNF inhibitor medication) with respect to their humoral responses to SARS-CoV-2 vaccination. The levels of neutralizing antibody titres were the primary outcome measures. They were recorded 6 ± 1 weeks after the second dose of the vaccine used (BioNTech/Pfizer, Moderna or AstraZeneca).

The study was organized and conducted in full accordance with the principles and criteria of good clinical practice [24, 25]. All patients enrolled gave written informed consent to participate in the study and agreed to the publication of any scientific results obtained. This study was approved by the Ethics Committee of the University Hospital of Würzburg, Würzburg, Germany (207/21-me). The organization and implementation of the study was in full accordance with the principles and criteria of ‘Good Clinical Practice’ (Declaration of Helsinki).

### Assessment of the immune response

The humoral immune response was the main outcome variable in our study and was assessed by determining titres of neutralizing antibodies against SARS-CoV-2. We used a quantitative ELISA test for IgG antibodies against the S1 antigen of SARS-CoV-2: Anti-SARS-CoV-2-QuantiVac ELISA (IgG); manufacturer: EUROIMMUN Medizinische Labordiagnostika AG, Lübeck, Germany.

### Statistical analysis

Sample size considerations referred to the following key points. We assumed two independent samples (patients with *vs*. without anti-TNF medication), a significance level of 5% and a statistical power of ≥80%, and we wanted to be able to detect an effect size of at least *d* = 0.6. Based on these considerations, the optimal sample size for two-sided testing was 90 subjects (i.e. ≥45 individuals per independent study subgroup: patients with TNF inhibitor *vs*. controls without any kind of immunomodulatory medication). Data handling and processing, statistical analyses and the creation of graphics and tables were performed using Microsoft Excel or SPSS software (German v.17.0.0), where appropriate [26]. Inferential tests were considered statistically significant at *P* < 0.05. Moreover, Pearson χ^2^ tests were applied to compare frequencies of categorical variables between independent subgroups according to therapy regimen. One-way ANOVA was used to test for mean differences in continuous variables between independent patient subgroups.

Given that the main outcome variable (titres of neutralizing antibodies) only reached ordinal data levels owing to laboratory-related ceiling effects, we used non-parametric inferential tests for independent samples in this case (Mann–Whitney *U* tests).

## Results

In our study, subgroups with (*n* = 45) and without (*n* = 62) anti-TNF medication did not differ in a statistically significant manner with respect to main sociodemographic and medical data. In particular, the frequencies of vaccines used were not significantly different when comparing the two study arms (*P* = 0.288; see Table 1). In the total sample, the distribution of vaccines used was as follows: BioNTech/Pfizer, 72.9%; AstraZeneca, 17.8%; and Moderna, 9.3%.

**Table 1.** Patient characteristics and relevant medical data (by patient subgroups with and without medication with TNF inhibitors) [22]

Characteristic	RA patients with anti-TNF medication	Reference group without anti-TNF medication	*P*-value
	(*n* = 45)	(*n* = 62)	
Age, years	61.3	64.3	0.182
Female sex, %	75.6	79.0	–
Male sex, %	24.4	21.0	0.670
Mean RA disease duration, years	13.6	0	n.a.
Seropositivity, %	84.4	0	n.a.
Prednisolone use, %	11.1	0	n.a.
Mean dose prednisolone, mg/day	4.50	0	n.a.
Diabetes, %	8.9	12.9	0.516
Mean GFR values, ml/min	79.33	81.26	0.626
Mean systolic BP, mmHg	142.96	143.00	0.995
Mean diastolic BP, mmHg	85.96	83.44	0.612
BioNTech/Pfizer, %	80.0	67.7	–
AstraZeneca, %	11.1	22.6	–
Moderna, %	8.9	9.7	0.288
SARS-CoV-2 IgG, BAU/ml	298.4	345.1	0.041
Maximum response (≥384 BAU/ml), %	62.2	77.4	–
Moderate response (176–383 BAU/ml), %	13.3	14.5	–
Low response (34–175 BAU/ml, %	17.8	8.1	–
Non-response (<34 BAU/ml), %	6.7	0	0.045

Seropositivity was defined as positivity for RF and/or ACPA.

BAU: binding antibody units; BP: blood pressure; GFR: glomerular filtration rate; SARS-CoV-2: severe acute respiratory syndrome coronavirus 2.

We found that the laboratory data showed significant ceiling effects in a large proportion of participants. In terms of neutralizing antibody levels, 76 of 107 study participants (71.0%) had a maximum antibody response of ≥384.0 binding antibody units (BAU)/ml. Beyond this upper limit, the assay was unable to discriminate any further. Given that these data were not normally distributed, we chose to analyse the antibody response statistically using non-parametric tests. We therefore performed Mann–Whitney *U* tests to compare vaccination-induced neutralizing antibody levels between independent subgroups of patients with and without TNF inhibitor medication. We used a four-point Likert scale that was largely self-explanatory and determined by the laboratory test used: no response, <34 BAU/ml; low response, 34–175 BAU/ml; moderate response, 176–383 BAU/ml; and maximal vaccine response, ≥384 BAU/ml.

**Figure 1.**
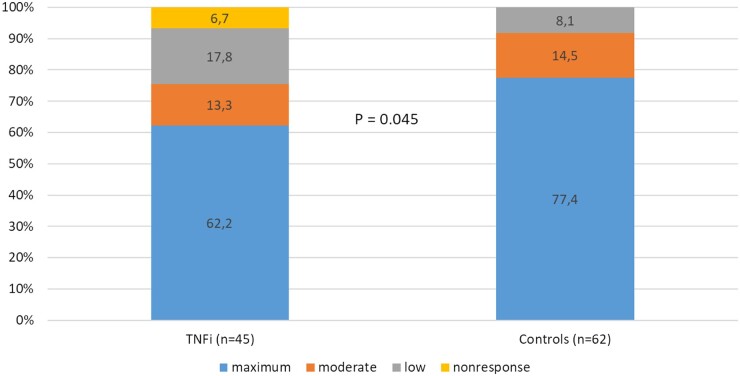
Titres of neutralizing IgG antibodies. Titres of neutralizing IgG antibodies against the S1 antigen of severe acute respiratory syndrome coronavirus 2 (SARS-CoV-2) depending on the use of TNF inhibitors (four-point Likert scale): non-response, <34 BAU/ml; low response, 34–175 BAU/ml; moderate response, 176–383 BAU/ml; and maximum response, ≥384 BAU/ml. Titres of neutralizing antibodies differed significantly between patients receiving TNF inhibitors (*n* = 45; 62.2% with maximum response) and control patients (*n* = 62; 77.4% with maximum response; *P = 0.045; *d* = 0.194). BAU: binding antibody units

We found that, overall, individuals treated with TNF inhibitors had a significantly reduced antibody response to SARS-CoV-2 vaccination (*P* = 0.045; Mann–Whitney *U* test; Table 1). Only 62.2% of patients on anti-TNF treatment showed a maximum response, whereas 77.4% of controls reached this level. This TNF inhibitor-induced reduction in humoral response was statistically significant (see above) and showed a clear and therefore probably clinically relevant effect size of *d* = 0.194. The associated results are shown in Fig. 1.

We also analysed whether there was a significant association between the antibody response and indicators of disease activity (DAS28 and CRP). Calculated non-parametric Spearman correlations were neither relevant (in terms of magnitude) nor statistically significant in our sample of RA patients (all respective correlation coefficients were <0.160, and all corresponding *P*-values were >0.590).

A subgroup of patients in the treatment arm received a combination of TNF inhibitors and MTX (17 of 45 patients, 37.8%). Our statistical analyses indicated that this factor (combined treatment with anti-TNF and MTX *vs*. anti-TNF as monotherapy) did not significantly affect the magnitude of the vaccine response (*P* = 0.214): 67.9% of monotherapy patients had a maximum vaccination response. This figure decreased to 52.9% in patients on combination therapy (anti-TNF and MTX). However, the subgroup sizes were not large enough to draw any general conclusions on the effect of combination treatment with anti-TNF and MTX. Originally, the study was not powered for this.

The influence of prednisolone use (*n* = 6) on vaccine response was not statistically significant in our anti-TNF cohort (*P* = 0.063). However, the sample size and statistical power for this sub-analysis were small and did not allow a definitive assessment of the effect of glucocorticoids in this context. In addition, the average daily dose of prednisolone was relatively low (4.50 mg). In total, 86.7% of patients treated with TNF inhibitors did not receive any glucocorticoid medication.

## Discussion

Vaccination against SARS-CoV-2 is still considered the decisive step against COVID-19. This is particularly important for patients with an inherently increased risk of infection, such as those with IMIDs. In a Canadian cohort, Widdifield *et al.* [27] showed very early that two vaccinations were effective in protecting against COVID-19 and prevented severe courses of infection in patients with RA, axial SpA, psoriasis or IBD. Regarding vaccination success, other factors, such as older age, have been identified as risk factors for a reduced immune response [28]. Simon *et al.* [29] were able to show that an underlying IMID itself influences the vaccine response. They also showed early evidence of differential effects of DMARDs on the immunogenicity of a vaccination. The fact that DMARDs can influence the humoral and cellular immune response has been shown for several vaccine-preventable diseases, such as pneumococci and influenza [21]. With regard to SARS-CoV-2, Syversen *et al.* [30] published respective data on patients with IMIDs. In addition to individuals with RA, the cohort included patients with axial SpA, PsA, Crohn’s disease or ulcerative colitis. Vaccination success, as measured by neutralizing antibodies to the receptor binding domain (RBD) of the SARS-CoV-2 spike protein, was assessed after two SARS-CoV-2 vaccinations in a prospective study design. Responders were seen in >90% of patients treated with MTX, TNF inhibitor monotherapy, ustekinumab, tocilizumab and vedolizumab. Response rates ranged from 80 to 90% in patients receiving combination TNF inhibitor therapy and were <80% in patients treated with JAK inhibitors (78%) or abatacept (53%). Patients had significantly lower median overall antibody levels than healthy controls. The results of their study, which was the largest in terms of cohort size in a long time, are essentially consistent with our data. We have also shown that the use of TNF inhibitors results in a significant attenuation of the humoral immune response. Our results are supported further by the work of Nemeth *et al.* [31] in RA patients and Venerito *et al.* [32] in patients with PsA.

In large studies of patients with IBD, Liu *et al.* [33] and Otten *et al.* [34] were also able to show an attenuation of the humoral immune response under treatment with the TNF inhibitor infliximab. As a result, an increased rate of breakthrough infections with SARS-CoV-2 has been reported [35]. TNF inhibitors can be used both as monotherapy and in combination with MTX in RA. This raises the question of the role of MTX as a combination partner of TNF inhibitors and its influence on the humoral vaccination response. We observed that the combination with MTX resulted in an additional reduction in neutralizing IgG titres, although this was not statistically significant. It is important to note, however, that we cannot draw a general conclusion from this owing to the small size of the subsamples with and without combination therapy in our study. Saad *et al.* [36] also demonstrated that TNF inhibitors attenuate immunogenicity in patients with axial SpA and confirmed that MTX in combination with TNF inhibitors had a major negative impact on the humoral vaccine response. Furer *et al.* [37] and Haberman *et al.* [38] were also able to show a significant reduction in the rate of seropositivity in patients treated with a combination of TNF-α inhibitors and MTX compared with those on TNF monotherapy. In view of the known effects of the underlying IMID on the humoral response, the heterogeneous patient cohorts in the work of Furer *et al.* should be noted, which is in contrast to our work. Le Moine *et al.* [39] also investigated the importance of combination therapy with DMARDs, including glucocorticoids. Importantly, all patients receiving monotherapy or a combination of csDMARDs without glucocorticoids seroconverted at significantly higher titres than patients receiving csDMARDs in combination with bDMARDs or tsDMARDs. The lowest seropositivity rate was for combination b/tsDMARDs, csDMARDs and glucocorticoids. The overall rate of seroconversion in our study was 94.1% in patients on combination therapy (anti-TNF and MTX). The proportion of patients on glucocorticoids was only 5.6% with a mean daily dose of 4.5 mg of prednisolone, hence no general conclusions could be drawn about the effect of glucocorticoids based on our data.

Differences in humoral and cellular immune responses depending on the vaccine used have also been shown previously in patients with IMIDs [40]. Le Moine *et al.* [39] found that the use of the AstraZeneca vector vaccine was associated with an overall lower SARS-CoV-2 IgG seropositivity rate compared with the mRNA vaccines. In our cohort, the AstraZeneca vector vaccine was used in addition to the available BioNTech/Pfizer and Moderna mRNA vaccines. However, the small number of cases in our cohort did not allow for further statistical evaluation of potential differential vaccination success depending on the type of vaccine used.

The main limitation of our study was the lack of data on the cellular response to vaccination. Nevertheless, the determination of neutralizing antibodies is currently the only widely used method for measuring vaccine response in routine clinical practice. There are also data suggesting a correlation between the humoral and cellular responses to vaccination [41]. However, the absence of seroconversion does not necessarily imply the absence of a vaccination response (e.g. in the form of a specific T cell response [17]). Another shortcoming is the use of different vaccines. To improve comparability, only vaccines that required at least two doses for baseline immunization were included. In order to determine the exact contribution of anti-TNF medication to the observed impaired immune response, an untreated control group of RA patients would have been required, which was not possible for ethical reasons.

The main strength of the study is the homogeneous composition of the patient population, exclusively consisting of RA patients. Moreover, the age range was comparable between patients and control subjects. In addition, there was a very low rate of glucocorticoid use, and the time of measurement of neutralizing antibodies was strictly limited to 6 ± 1 weeks after the second vaccination.

## Data Availability

The data underlying this article will be shared on reasonable request to the corresponding author.
